# Risk Perception of COVID-19 Infection and Adherence to Preventive Measures among Adolescents and Young Adults

**DOI:** 10.3390/children7120311

**Published:** 2020-12-21

**Authors:** Xin Yu Yang, Rui Ning Gong, Samuel Sassine, Maxime Morsa, Alexandra Sonia Tchogna, Olivier Drouin, Nicholas Chadi, Prévost Jantchou

**Affiliations:** 1 CHU Sainte-Justine Research Center, Montréal, QC H3T 1C5, Canada; xin.yu.yang@umontreal.ca (X.Y.Y.); rui.ning.gong@umontreal.ca (R.N.G.); samuel.sassine@umontreal.ca (S.S.); soniaalexandra.tchogna@gmail.com (A.S.T.); o.drouin@umontreal.ca (O.D.); nicholas.chadi.hsj@ssss.gouv.qc.ca (N.C.); 2 Faculty of Medicine, Université de Montréal, Montréal, QC H3T 1J4, Canada; maximemorsa@hotmail.com; 3 Laboratory of Education and Health Practices (UR 3412), University Sorbonne Paris Nord, 93017 Bobigny, France; 4 Division of General Pediatrics, Department of Pediatrics, CHU Sainte-Justine, Montréal, QC H3T 1C5, Canada; 5 Department of Social and Preventive Medicine, School of Public Health, Université de Montréal, Montréal, QC H3N 1X9, Canada; 6 Division of Adolescent Medicine, Department of Pediatrics, CHU Sainte-Justine, Montréal, QC H3T 1C5, Canada; 7 Division of Gastroenterology, Department of Pediatrics, CHU Sainte-Justine, Montréal, QC H3T 1C5, Canada

**Keywords:** SARS-CoV-2, COVID-19, pandemic, risk perception, preventive measures, adolescents, young adults

## Abstract

To explore factors influencing adolescents and young adults’ (AYAs) risk perception of COVID-19 and adherence to public health measures, we conducted a cross-sectional online survey of AYAs (14–22 years old) from Quebec (Canada) recruited through school and community partners in April 2020 during the first wave of the COVID-19 pandemic. The study included 3037 participants (mean age = 17.7 years, 74.6% female). AYAs had higher mean (standard deviation (SD)) risk perception of COVID-19 for their relatives (8.2 (1.9)) than for themselves (5.6 (2.6)) (*p* < 0.001). Factors associated with higher risk perception included higher disease knowledge (adjusted odds ratio (aOR) 1.06, 95% CI 1.01–1.11), presence of chronic disease (aOR 2.31, 95%CI 1.82–2.93) and use of immunosuppressants (aOR 2.53, 95%CI 1.67–3.87). AYAs with a higher risk perception (aOR 1.06, 95%CI 1.02–1.10) those wishing to help flatten the disease curve (aOR 1.18, 95%CI 1.12–1.25) or to protect their family/friends (aOR 1.14, 95%CI 1.05–1.24) were more likely to engage in preventive behaviors. Self-perceived risk and desire to protect others were significantly associated with adherence to preventive measures among youth. These findings may help inform public health messaging to AYAs in the current and future pandemics.

## 1. Introduction

The majority of pediatric cases of coronavirus disease 2019 (COVID-19) are mild to moderate [1,2], with morbidity and mortality concentrated in individuals with advanced age and chronic medical conditions [3,4]. Although adolescents and young adults (AYAs) experience fewer complications from the disease, they may represent a risk to vulnerable populations by acting as carriers of the virus [5,6]. Recent estimates suggest that the probabilities to be asymptomatic carriers, from whom viral transmission is possible [7,8], are 6.3% and 14.3% in adolescents and young adults, respectively [9]. In an effort to limit community transmission of the virus, strict hygiene and social distancing measures, including school and workplace closures were issued in several countries. The respect of these measures is critical for them to be effective [10].

Evidence suggests that adherence to preventive measures is influenced by real or perceived risk of infection whereby a lower perceived risk leads to lower adherence [11,12,13,14]. AYAs are usually a population with a lower perception of risk than other age categories [15] which may in fact be explained by the lower morbidity and mortality seen in this age group.

While the presence of chronic medical conditions and exposure to immunosuppressant medication may increase the risk and complications of COVID-19 infection, AYAs’ self-perceived risk may remain low because of their young age [3,4]. Other than underlying medical related risk factors, previous studies have also linked older age, female gender, and better disease knowledge to higher risk perception [11,14,15]. In the context of the current pandemic, it is imperative to understand AYAs’ risk perception and underlying factors as they could be targets for future public health messages.

AYAs’ adherence to preventive measures such as social distancing, quarantine, and isolation may interfere with the ability of maintaining an active social life, an important resource to maintain their wellbeing [16,17]. Nevertheless, several studies have demonstrated that in the context of epidemics, which tend to have a disproportionate impact on vulnerable populations, adherence to preventive measures by AYAs may be more motivated by community rather than personal motives or self-interest [18,19]. Therefore, in addition to risk perception, other factors may affect adherence to preventive measures in AYAs, such as community attachment, empathy [20,21], and altruism [18]. Altruism has been described as a driver for other preventive behaviors in the context of epidemics. For example, evidence suggests that altruism has the potential of impacting vaccination decisions by prioritizing community over self-interest in adults [19]. In addition, it has been shown that altruism develops in adolescence along with a feeling of solidarity with peers and members of specific networks, but it remains unclear whether adolescents are more or less altruistic than adults considering that they may become less concerned about justice and fairness but are also more idealistic. Therefore, it will be important to assess how the level of altruism in AYAs influences the level of adherence to preventive measures.

The primary aim of this study was to assess AYAs’ risk perception of COVID-19 infection and factors influencing this risk perception. The secondary aim was to assess the determinants of AYAs adherence to preventive measures, including risk perception and motivating factors such as altruism. We hypothesized that high risk perception and motivational factors involving altruism would be positively associated with adherence to preventive measures.

## 2. Materials and Methods

### 2.1. Study Design, Participants, Procedure

We conducted a cross-sectional online study of AYAs aged 14 to 22 years-old, living in Quebec, Canada from 2 April to 28 April 2020, a period of time during which public measures such as school closures, home confinement and travel restrictions were put in place to contain the spread of the virus. Data were collected through a self-administered French or English electronic questionnaire depending on participants’ preferences. Participants were recruited through email invitation from Quebec school boards, colleges and universities. To increase representativity of the general AYA population in Québec and limit potential selection bias, multiple organizations interacting with minority populations and youth with chronic illnesses were approached by email, namely: ethno-cultural centers, community centers and scientific societies (Canadian Cancer Society, Coeliac Quebec, Crohn Colitis Canada, Diabetes Canada). Campaigns were also carried out on social networks, regional newspapers, as well as on the province largest pediatric hospital’s website and popular social media platforms. Among the 670,000 AYAs living in the province of Quebec, around 10% of young people between age of 12 to 24 years have a chronic disease (including inflammatory bowel diseases, arthritis, asthma, cancer, cardiovascular diseases, chronic respiratory diseases, diabetes mellitus, and mood disorders) and 27% belong to a visible minority as defined by the Employment Equity Act [22,23,24]. A sample size of at least 500 participants was required to ensure appropriate representativeness of AYAs with chronic diseases and ethnical minority with a representativeness margin of error of 5% and a confidence interval of 95%. 

The study was approved by the research ethics board of Sainte-Justine Hospital research center (2020–2864). All participants provided consent by accepting to answer the questionnaire. Three annual subscriptions to a video streaming service were drawn at random among study participants as an incentive to participate. 

### 2.2. Measures

The questionnaire design was based on an extensive review of existing questionnaires developed during past MERS, SARS, and influenza pandemics [14,25,26,27,28] and adapted for COVID-19. The questionnaire was developed in English and French by bilingual experts (pediatricians, epidemiologists, public health specialists) with support of an external reviewer. The questionnaire was then validated with 38 participants representative of the target population. Data was analyzed to ensure feasibility, comprehension, and face validity. The Cronbach’s alpha coefficient (α) for the overall survey was 0.72.

The questionnaire was divided into six sections with all the questions being mandatory:

1. General demographic information including age, gender, ethnicity, education, work status before and during the pandemic, language that the questionnaire was answered in, as well as data on pre-existing chronic diseases or medication.
2. Perceived level of information about COVID-19 was measured with a single item (well-informed about COVID-19—yes/no). AYAs also reported which sources they used for COVID-19 related information among social media, broadcasting media, specialized internet resources, conversations with friends/family or others.
3. Objective knowledge of COVID-19 manifestations, disease transmission and recommended preventive attitudes were measured using twenty true/false questions, yielding a total knowledge score ranging from 0 to 20, reported as a percentage of correct answers (0–100%).
4. Adherence to preventive measures recommended by public health authorities were measured with 4 items (Cronbach’s α = 0.72). AYAs reported how often they washed their hands, avoided group gatherings, reduced unnecessary use of public transportation and avoided public places, using 5-point Likert scales from *Never* to *Always.* Participants who answered that they *Always* followed the four recommendations were classified as having *high adherence* to preventive measures while others were classified as having *low adherence*;
5. Perception of the risk of becoming a person with COVID-19 was assessed with 2 items (Cronbach’s α = 0.79) in which AYAs reported the perceived risk of COVID-19 infection for themselves and for their family/friends. Responses were given on an 11-point Visual Analog Scale (VAS) from 0 (*no risk at all*) to 10 (*extremely important risk*).
6. Assessment of factors motivating preventive behaviors included four items (Cronbach’s α = 0.64). Two items related to altruistic motivations: (1) Desire to help to flatten the curve of disease (i.e., reduce the number of new COVID-19 cases)*;* (2) Desire to protect friends/family who are more at risk. Two items were related to personal motives: (3) perceived negative impact on their social life and (4) feeling of not being concerned by COVID-19. All the responses in this section were recorded by a 11-point VAS from 0 (*I don’t agree at all*) to 10 (*I fully agree*).

### 2.3. Data Analysis

We first conducted descriptive statistics, with data reported as mean and standard deviation for continuous variables with normal distribution, or medians and interquartile ranges for continuous variables with skewed distributions. Categorical variables were reported as frequencies and percentages.

A multivariable ordinal logistic regression was conducted using risk perception as the dependent variable and a multivariable logistic regression analysis was conducted using adherence to preventive measures as the dependent variable. The regression models included the following independent variables: age (in years), gender (male/female), language that the questionnaire was answered in (English/French), Current school program (Natural sciences, Health sciences, Art and social sciences, Early childhood care and education), ethnicity (Afro-Caribbean, Asian, Caucasian, Central and South America, Native American, Middle East), work status before the pandemic (Yes/No), COVID-19 related knowledge, presence of an underlying chronic disease (Yes/No), and exposure to immunosuppressant medication (Yes/No). The risk perception and motivating factors were additionally included in the model assessing adherence to preventive measures. All analyses were performed with SAS statistical software, version 9.4. 

## 3. Results

### 3.1. Demogaphic Data

A total of 3037 participants completed the survey. Mean participant age was 17.7 years (standard deviation (SD) 2.7 years) and 2266 (74.6%) were female (Table 1). Except for gender (female to male ratio of 3:1), the study was representative of the AYA population of the province of Québec [22]. Eleven percent (*n* = 348) had at least one chronic disease. Among them, 105 (3.4%) reported currently taking immunosuppressants.

### 3.2. Objective Knowledge of COVID-19

The mean knowledge score was 80.0% (SD 10.0%). On average, females had a higher knowledge score of 80.5% (SD 9.9%) compared to males, who had an average score of 79% (SD 10.2%, *p* < 0.001). Similarly, AYAs living with a chronic disease had a higher disease knowledge score than their healthy peers, at 81.3% (SD 10.7%) and 80% (SD 9.8%) respectively (*p* = 0.04). Ninety percent of AYAs considered themselves well-informed about COVID-19. AYAs who considered themselves well-informed reported higher use of reliable sources such as government press conferences and websites, and lower use of social media platforms (Table S1, online only).

### 3.3. Risk Perception

**Figure 1:**
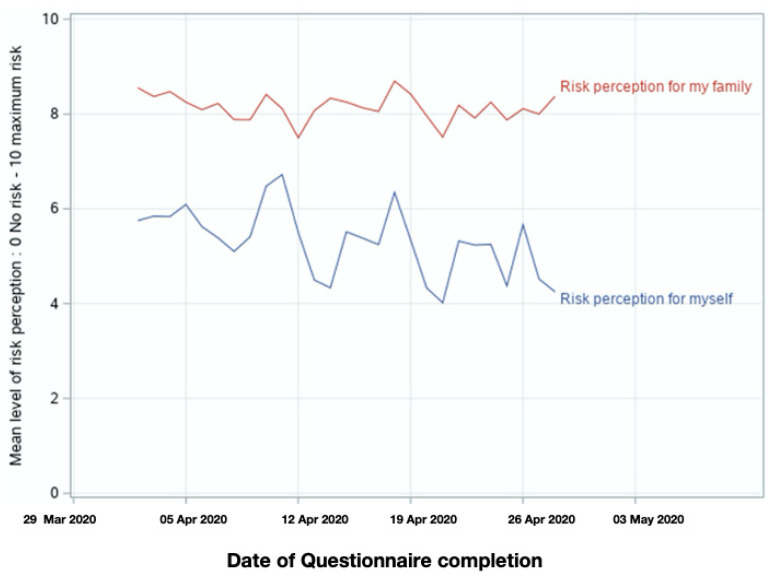
Variation of the mean level of participants’ risk perception for themselves and for their family.

On average, AYAs evaluated the perceived risk of COVID-19 infection at 5.6/10 (SD 2.6) for themselves and 8.2/10 (SD 1.9) for their family/friends (*p* < 0.001). AYAs living with a chronic disease perceived higher risk of COVID-19 infection for themselves than their healthy peers at 7.0/10 (SD 2.5) and 5.4/10 (SD 2.6) respectively (*p* < 0.001). In addition, AYAs, using an immunosuppressant medication had a higher perceived risk of infection of 7.7/10 (SD 2.2) vs. a risk perception of 6.7/10 (SD 2.6) for those who did not take any immunosuppressant medication (*p* < 0.001). Over the four weeks of data collection, the perceived risk of infection for AYAs themselves decreased modestly (as shown on Figure 1), with a difference of 0.73/10 between the mean perceived risk reported during week 1–2 of data collection and the mean perceived risk reported during week 3–4.

Risk perception significantly differed based on demographic factors and disease knowledge in multivariate analyses. Using ordinal logistic regression, we found that higher disease knowledge score (adjusted odds ratio (aOR) 1.06, 95%CI 1.01–1.11), presence of chronic disease (aOR 2.31, 95%CI 1.82–2.93) and use of immunosuppressants (aOR 2.53, 95%CI 1.67–3.87) were associated with a higher risk perception of COVID-19 infection but not age nor gender (Table 2).

### 3.4. Adherence of Preventive Measures and Motivators

In total, 1823 (60.0%) respondents reported high adherence to the four preventive measures: respect of all four measures including washing their hands, avoiding group gatherings, reducing unnecessary use of public transportation and avoiding public places. The rate of adherence to preventive measures was similar for the subset of AYAs living with chronic disease (61.3% of adherence) and those taking an immunosuppressant medication (63.8% of adherence).

In multivariable analyses, female gender (aOR 1.29, 95%CI 1.08–1.55) and higher risk perception (aOR 1.06, 95% CI 1.02–1.1) were associated with higher odds of adherence to preventive measures (Table 3. Likewise, higher desire to flatten the curve of disease (aOR 1.18, 95%CI 1.12–1.25) or to protect their family/friends (aOR 1.14, 95% CI 1.05–1.24; *p* = 0.002) were also associated with a greater level of adherence to preventive measures (Table 3).

The negative impact of containment on AYAs life and the feeling of not being concerned by the COVID-19 pandemic were not associated with lower adherence to preventive measures (Table 3 and Table S2, online only).

## 4. Discussion

The present study collected data from a large sample of AYAs from the province of Quebec. Overall, AYAs perceived a low risk of COVID-19 infection for themselves and a high risk for others. In this study, higher risk perception was associated with better adherence to preventive measures. The presence of baseline risk factors (chronic disease or use of immunosuppressants) and a better knowledge of the disease were associated with a higher risk perception. Other motivating factors enhancing adherence to preventive measures included flattening the curve of disease and protecting family/friends who are more at risk. These findings have implications for the elaboration of public health messages.

First, improving disease knowledge may be an effective strategy to enhance individual risk perception. In the context of a pandemic where information is constantly updated, there is a need for clear educational messages that are accessible and useful for AYAs [29]. Otherwise, AYAs may not be able to evaluate the risks associated with COVID-19 infection and to apply appropriate health measures to protect themselves and those around them [30].

Higher risk perception was associated with higher adherence, but with an effect size that was smaller compared to other motivating factors linked to community attachment such as the desire to help flattening the curve of disease or protecting family/friends who are more at risk. This is consistent with other studies showing that, for both adolescents and adults, the main motivations for social distancing include social responsibility and desire to protect others ([31], Coroiu, 2020 #6246, Oosterhoff, 2020 #6214). These results are further confirmed by another study conducted among Italian adults that showed that in the context of COVID-19, civic attitudes, including the feeling of involvement in the community, have an impact on adherence to preventive measures [32]. Therefore, promoting altruism and community attachment could be a potential strategy to enhance AYAs’ uptake of current preventive measures, and could also encourage vaccination once it will be available. Further, more generally, adapted public health interventions emphasizing social responsibility during the current pandemic could enhance AYAs’ autonomy and allow them to play a role in awareness communications to peers and family. This approach is valuable as it has been demonstrated that adolescents’ health behaviors are greatly influenced by their peer social network [33].

Neither the negative impact of containment on AYAs’ social life nor the feeling of not being concerned by the COVID-19 pandemic had an effect on AYAs’ adherence to preventive measures, suggesting that the altruistic desires to protect others who are more vulnerable and to help control disease transmission represent sufficient motivators for preventive behavior changes despite the perceived negative impact. These results suggest that future sensibilization messages or public health campaigns could potentially emphasize flattening of the curve of disease and protecting family/friends who are more at risk as main motivators to encourage AYAs to adhere to preventive measures. This could be done by tailoring messages to the target audience, by increasing personal relevance, by storytelling or by using social media influencers to communicate theses public health recommendations to AYAs [34]. This approach could be valuable as another study confirmed that AYAs often value actions leading to solidarity and altruism, thus communicating these messages to the general population of AYAs via means of mass media and popular social media platforms might be effective [18]. This perspective is also part of the World Health Organization’s “One Health” approach highlighting the interconnection between personal health and environment [35]. 

We had anticipated that AYAs with increased vulnerability (chronic illness or immunosuppression) would express an increased perceived risk leading to higher adherence to preventive measures. However, our results showed that the rate of adherence to preventive measures were rather similar between AYAs living with chronic diseases or taking immunosuppressant medication as compared to their healthy peers. One hypothesis explaining this finding could be that adolescents recognized a potential vulnerability associated with their medical condition, but were not worried since public health messages were directed, during the first wave of the pandemic, mainly towards the protection of the elderly. Therefore AYAs, feeling neglected by public health messages, despite the recognition of their potential vulnerability, may not have considered adherence to preventive measures as a priority. It is therefore crucial to raise risk awareness among AYAs living with chronic illnesses or taking immunosuppressant medication but having low perceived risk, without adding to their level of anxiety [36]. In this context, an individualized approach including education and guidance by health care professionals with closer follow-ups may be considered.

Our results should be interpreted considering certain limitations. First, our sample, although very large, is approximately three-quarters female and one-quarter male, which limits the generalizability of our findings, though we did control for gender in our multivariate analyses. Recruitment for our survey was voluntary, and potentially skewed towards individuals with higher levels of education and/or concerns about the COVID-19 pandemic. Second, we recognize that many other unmeasurable factors influencing risk perception and adherence to preventive measures may be present. Finally, given the pandemic’s rapidly evolving state, it is likely that the respondents’ answers could have changed during the data collection period (Figure 1). However, the decrease in mean risk perception scores with time although statistically significant, had a modest effect size of 0.73 points on the scale (0–10). The cross-sectional nature of this study did not allow us to determine the impact of measures being implemented as the study was undertaken on individual changes in perception and behavior. Although our current results reported that AYAs adhered to preventive measures regardless of the negative impacts of confinement during the beginning of the first wave of the COVID-19 pandemic, this may change as the pandemic continues. Conducting a longitudinal cohort study to improve understanding of factors influencing changes in risk perception and adherence to preventive measure is the logical next step for our work and is currently underway. 

## 5. Conclusions

Though less severely affected than older individuals by the COVID-19 pandemic, AYAs can play an important role in the societal effort to reduce disease transmission by adopting preventive behaviors. Our study showed that youth are sensitive to concepts of social responsibility and value protection of vulnerable family members or friends. These findings have important clinical and public health implications for public health messaging in the wake of potential subsequent waves of the pandemic.

## Figures and Tables

**Table 1:** Participant characteristics ^a^.

**Total, No. (%)**	3037 (100)
Age, median (IQR), years	17 (15–20)
**Gender, No. (%)**	
Male	740 (24.4)
Female	2266 (74.6)
Other	31 (1.0)
**Language used to answer the questionnaire, No. (%)**	
French	2905 (95.6)
English	132 (4.4)
**Education, highest diploma obtained, No. (%)**	
Elementary	1144 (37.7)
Secondary	1055 (34.7)
College	693 (22.9)
University	137 (4.5)
Other	4 (0.1)
None	4 (0.1)
**Current school program, No. (%) (*n* = 1569) ^b^**	
Natural sciences (Sciences, mathematics, and engineering)	347 (22.1)
Art and social sciences	639 (40.7)
Early childhood care and education	83 (5.3)
Health Sciences	461 (29.4)
Other	39 (2.5)
**Ethnicity, No. (%)**	
Afro-Caribbean	158 (5.2)
Asian	161 (5.3)
Caucasian	2274 (75.3)
Central and South America	157 (5.2)
First Nations	105 (3.5)
Middle East	154 (5.1)
Other	12 (0.4)
**Employed before the pandemic (full time or part-time), No. (%)**	1441 (47.4)
**Current work status, No. (%) (*n* = 1441) ^c^**	
Work less	131 (9.1)
Work more	95 (6.6)
Work the same	310 (21.5)
Work from home	104 (7.2)
Stopped working	801 (55.6)
**Disease knowledge score, No. (%) ^d^**	
Under 50%	10 (0.3)
50–74%	627 (20.6)
75–89%	1679 (55.3)
More than 90%	721 (23.7)
**Know someone with COVID-19, No. (%)**	556 (18.3)
Self	15 (0.5)
Family	165 (5.4)
Friends	258 (8.5)
Acquaintance	139 (4.6)
**Know someone who died from COVID-19, No. (%)**	54 (1.8)
Family	23 (0.8)
Friends	13 (0.4)
Acquaintance	18 (0.6)
**Chronic disease, No. (%)**	348 (11.5)
**Currently taking medication, No. (%)**	255 (8.4)
**Currently taking Immunosuppressant medication, No. (%)**	105 (3.4)
**Strictly follow preventive measures, No. (%) ^e^**	1823 (60.0)
Risk perception score, mean (DS)	5.56 (2.6)

^a^ All percentages are column percentages, ^b^ Among participants who have a specific program, ^c^ Among participants who worked before the pandemic, ^d^ Disease knowledge score represents a participant’s cumulative score on 20 true or false questions about COVID-19 disease characteristics, ^e^ Defined as self-reported respect of all following preventive measures: regular handwashing, avoiding group gathering, reducing unnecessary use of public transport, and avoiding public places.

**Table 2:** Factors associated with high COVID-19 disease risk perception ^a,b^.

	Adjusted Odds Ratio	95% Confidence Interval	*p* Value
Current school program ^c^			
Early childhood care and education program in college or in university	1.70	(1.12–2.59)	0.01
Disease knowledge score ^d^	1.06	(1.01–1.11)	0.02
Chronic disease	2.31	(1.82–2.93)	<0.001
Immunosuppressant medication	2.53	(1.67–3.87)	<0.001

^a^ An ordinal regression model has been used for this analysis and adjusted for: age, gender, ethnicity, work status before the pandemic, current school program, having a family member or friend with COVID-19, language used to answer this questionnaire, ^b^ Risk defined by a participant’s level of perceived risk of COVID-19 infection for themselves on an 11-point Visual Analog Scale from 0 to 10, ^c^ Participants in each program are compared with the rest of the cohort that does not study in this program, ^d^ For each point of score, total of 20 (disease knowledge score represents a participant’s cumulative score on 20 based on true or false questions about COVID-19 disease characteristics).

**Table 3:** Factors associated with the observance of preventive measures ^a,b^.

	Adjusted Odds Ratio	CI (95%)	*p* Value
Age	0.97	(0.93–1.01)	0.10
Female gender	1.29	(1.08–1.55)	0.006
Work before pandemic	0.79	(0.67–0.93)	0.004
Current school program ^c^			
Arts and social sciences	1.55	(1.21–1.98)	<0.001
Health sciences	1.86	(1.40–2.47)	<0.001
Disease knowledge score ^d^	1.03	(0.99–1.08)	0.12
Language used to answer this questionnaire			
French	1	1	0.003
English	1.94	(1.25–3.02)	
Higher perceived risk of COVID-19 infection ^e^	1.06	(1.02–1.10)	0.003
Motivators for taking preventive measures			
To flatten the curve	1.18	(1.12–1.25)	<0.001
To protect family and friends	1.14	(1.05–1.24)	0.002

^a^ Adjusted for: ethnicity, school program, having a family member or friend with COVID-19, negative motivators for taking protective measures (feeling of not being concerned, negative impact on social life), ^b^ Defined as self-reported respect of all following preventive measures: regular handwashing, avoiding group gathering, reducing unnecessary use of public transport, and avoiding public places, ^c^ Participants in each program are compared with the rest of the cohort that does not study in this program, ^d^ For each point of score, total of 20 (disease knowledge score represents a participant’s cumulative score on 20 based on true or false questions about COVID-19 disease characteristics), ^e^ Risk defined by a participant’s level of perceived risk of COVID-19 infection for themselves on an 11-point Visual Analog Scale from 0 to 10.

## Supplementary Materials

The following are available online at https://www.mdpi.com/2227-9067/7/12/311/s1, Table S1: Associations between perceived level of information, sources of information and overall level of knowledge about COVID-19, Table S2: Motivations for taking preventive measures against COVID-19.
