# Supplementary material for: Risk Perception of COVID-19 Infection and Adherence to Preventive Measures among Adolescents and Young Adults
Source: Children (Basel). 2020 Dec 21;7(12):311. doi: 10.3390/children7120311 (PMC7766485; doi:10.3390/children7120311)
Supplement: Supplementary file 1 [file children-07-00311-s001.pdf]

Table S1. Associations between perceived level of information, sources of information and overall level of knowledge about COVID-19 [a.]

	**Not well informed (*n* = 291)**	**Well informed (*n* = 2746)**	***P* Value**
Disease knowledge score, No. (%) [b]			
< 50%	3 (1.0)	7 (0.2)	.01
50%–74%	70 (24.0)	557 (20.3)	
75%–89%	166 (57.0)	1513 (55.1)	
≥ 90%	52 (17.9)	669 (24.4)	
Sources of information, No. (%) [c]			
Email from school	34 (11.7)	487 (17.7)	< .001
Family	90 (30.9)	1142 (41.6)	.01
Government press conference	85 (29.2)	1429 (52.0)	< .001
Other governmental source	20 (6.9)	364 (13.3)	< .001
Health care organization	14 (4.8)	312 (11.3)	< .001
Healthcare professional	14 (4.8)	326 (11.9)	< .001
TV or radio	98 (33.7)	1497 (54.5)	< .001
Facebook	35 (12.0)	398 (14.5)	.01

a. All percentages are column percentages, b. Disease knowledge score represents a participant's cumulative score on 20 true or false questions about COVID-19 disease characteristics, c. Participants could select more than one source of information.

Table S2. Motivations for taking preventive measures against **COVID-19** [a]

**No. (%)**	**Low respect for preventive measures (*n* = 1214)**	**High respect for preventive measures [b] (*n* = 1823)**	***P* Value**
Positive motivators			
Want to help to flatten the curve	1174 (96.7)	1805 (99.0)	< .001
Want to protect family and friends	1200 (98.8)	1818 (99.7)	.003
Negative motivators			
Negative impact of confinement on social life	863 (71.1)	1249 (68.5)	.13
Do not feel concerned by the COVID19 pandemic	245 (20.2)	262 (14.4)	< .001

a. All percentages are column percentages, b. Defined as self-reported respect of all following preventive measures: regular handwashing, avoiding group gathering, reducing unnecessary use of public transport, and avoiding public places.
